# Long-term dispersion of river gravel in a canyon in the Atacama Desert, Central Andes, deduced from their ^10^Be concentrations

**DOI:** 10.1038/s41598-019-53806-x

**Published:** 2019-11-28

**Authors:** Sébastien Carretier, Vincent Regard, Laëtitia Leanni, Marcelo Farías

**Affiliations:** 1 GET, Université de Toulouse, CNRS, IRD, UPS, Toulouse, France; 2 Aix Marseille Univ, CNRS, IRD, Coll France, CEREGE, Aix-en-Provence, France; 3 Department of Geology, FCFM, University of Chile, Santiago, Chile

**Keywords:** Geomorphology, Sedimentology

## Abstract

Intense storms or earthquakes in mountains can supply large amounts of gravel to rivers. Gravel clasts then travel at different rates, with periods of storage and periods of displacement leading to their downstream dispersion over millennia. The rate of this dispersion controls the long-term downcutting rate in mountainous rivers as well as the grain-size signature of climate and tectonic variations in sedimentary basins. Yet, the millennial dispersion rates of gravel are poorly known. Here, we use ^10^Be concentrations measured in individual pebbles from a localized source along a 56 km-long canyon in the Central Andes to document the distribution of long-term gravel transit rates. We show that an inverse grain-size velocity relationship previously established from short-term tracer gravel in different rivers worldwide can be extrapolated to the long-term transit rates in the Aroma River, suggesting some universality of this relationship. Gravel are also dispersed by large differences in the mean transport rates independent of gravel size, highlighting that some gravel rest at the river surface over tens of thousands of years. These different transport rates imply a strong spreading of the gravel plumes, providing direct proof for the long-term river buffering of sediment signals between mountainous sources and sedimentary basins. The inferred distribution of residence times suggests the first evidence of anomalous diffusion in gravel transport over long timespans.

## Introduction

Gravel dispersion in rivers is inherently a stochastic process^1^: each gravel clast travels at its own velocity, and can be stored temporarily during different periods at depth or in lateral deposits. When gravel accumulates, the vertical erosion of bedrock river beds is reduced, whereas the impact of moving gravel can boost river incision^2^. Furthermore, when an initial pulse of gravel is generated in a mountain catchment through renewed tectonic activity or climate change, it spreads downstream and mixes with previously detached gravel^3^. Thus, different periods of large sediment supply in the mountainous source may not be recorded in distant sedimentary basins^4^. Moreover, if the gravel clasts are stored and recycled several times, this means that it takes a particularly long time for them to reach the basin, and then the age of the gravel deposit may be significantly younger than the climatic or tectonic change in the source, potentially biasing the dating of source variations from the stratigraphic record^5,6^. The lithological composition of gravel in basins is also used to infer the denudation history of mountain ranges, but this approach is significantly complicated by repeated and long periods of sediment storage and recycling^7^, which are difficult to quantify^6,8^. The downstream spreading of gravel plumes is partly taken into account by models where the downstream transport of sediment is described by a diffusion equation^4,9,10^, but the diffusive nature of long-term river sediment transport has never been established from the dispersion of traced particles. Furthermore, recent experimental and field data have shown that sediment transport may not follow a diffusion equation on monthly or yearly timescales^11–16^. Extrapolating these findings to longer timescales is uncertain because the distribution of storage periods in floodplains is unknown^17^. Actually, most of the uncertainty lies in the lack of data to trace the transport of sediment over millennial timescales. Uranium-series^18,19^, cosmogenic nuclides^20–22^ and tracer thermochronology^23^ have provided evidence for very long transport times of several hundreds of thousands of years for fine sediment, even in active orogens such as Taiwan^19^ or New Zealand^23^. Nevertheless, the distribution of gravel residence times in river system, which is key to characterising the gravel transport dynamics, remains unknown. Only one study has recently inferred a distribution of large minimum gravel residence times in a modern river in the Great Plains (USA) using ^21^Ne concentrations in individual pebbles^8^.

The potential for using cosmogenic nuclides to document long-term (1–100 ka) sediment transport has long been recognised^24,25^. Pioneer works used the downslope increase in the cosmogenic nuclide concentration in sediments to quantify their mean transport rates on hillslopes^26–29^, and on desert piedmonts^20,21^. It is theoretically possible to quantify the distribution of gravel transport rates^30,31^, but this requires a single source of some particular lithology within a catchment. Then, by sampling the gravel from that particular lithology at different places along the river, it is possible to obtain the distribution of the ^10^Be concentrations in individual gravel, from which residence times between the source and sampling point can be estimated^30,31^. We followed this strategy in the Aroma River in the Central Andes.

**Figure 1:**
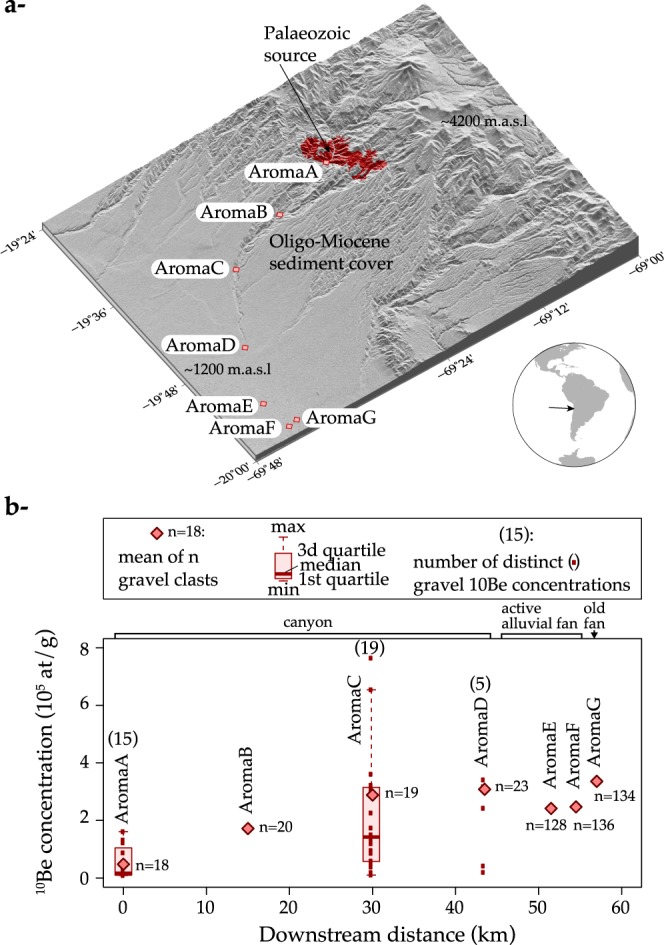
(**a**) Simplified geological map around the Aroma canyon (elevation data from SRTM 1 arc seconds digital elevation model^88^). The Palaeozoic gneiss body is exhumed in an erosion window below oligo-miocene sediment^84^. (**b**) Gravel clasts from this gneissic source were gathered downstream (cf. photos in Supplementary Fig. S1) and their ^10^Be concentrations were measured (analytical uncertainty smaller than the symbol size - Supplementary Table S1). The mean ^10^Be concentration at AromaG corresponds to an older fan surface sampled to verify the consistency of the ^10^Be concentration.

The Aroma catchment is located in Northern Chile on the western edge of the Altiplano plateau. The main river of the Aroma catchment incises into Neogene deposits forming a 56 km-long canyon ending in an alluvial fan^32^. Most of the rainfall comes from the catchment head during the summer months. Flash floods and debris flows occur during El Niño/La Niña events with return periods between several years to several centuries^33,34^. A Palaeozoic meta-sedimentary gneissic body is exhumed at the entrance of the canyon by a thrust and supplies gravel to the river via shallow landslides and detachment from the bedrock surface (Fig. 1). During this process and afterwards along the river, ^10^Be atoms are produced in gneissic pebbles when they reside at and near the surface (at a depth of 1 m, the ^10^Be production rate is ~20% of the one at the surface).

Our approach is to use the ^10^Be concentration in individual gneissic gravel clasts to infer their different residence times from their gneissic source on the hillslopes. Beginning at the location of the Palaeozoic gneiss source (AromaA), we sampled gneissic gravel clasts at seven locations (stations) along the Aroma River (AromaA to G). The last and more distal station AromaG corresponds to an alluvial surface above the more recent incised channel, and is therefore expected to be older with higher ^10^Be concentrations due to post-deposit exposure. Our previous theoretical study suggested that the best procedure to correct for the possible effect of pebble abrasion on the downstream ^10^Be concentration evolution was to sample the coarsest fraction of the river bed^31^. Based on this guideline, we sampled the coarsest sediment that we observed at each station, gathering gravel clasts from the river bed within a perimeter of approximately 10 m by 10 m. Although we did not measure the grain size distribution of the river bed, we observed a downstream decrease in maximum clast size, with the largest angular boulders reaching 1 m at AromaA, whereas the largest clasts corresponded to small rounded pebbles measuring several centimetres at the most distal station. Since it would be practically impossible to handle numerous whole boulders and cobbles, we collected a piece of rock (~0.5 kg and <5 cm thick) at the top surface of the cobbles (diameter *D* > 0.06 m - Supplementary Fig. S1). For smaller clasts (*D* < 0.06 cm), we took the whole clast.

In total, we sampled 478 clasts. We obtained the mean ^10^Be concentration of a population of clasts for seven river stations and the ^10^Be concentrations of 40 individual clasts (Fig. 1). To obtain the mean ^10^Be concentrations, we crushed the sampled rocks and mixed them by taking the same weight of material from each individual clast. The seven mean ^10^Be concentrations correspond to a mix of 18 angular cobble pieces at AromaA (*D* ∈ [0.11, 0.73] m), 20 rounded pebble pieces at AromaB (*D* ∈ [0.14, 0.2] m), 19 rounded pebble pieces at AromaC (*D* ∈ [0.11, 0.21] m), 23 rounded pebble pieces at AromaD (*D* ∈ [0.10, 0.24] m), 128 rounded small pebbles at AromaE (*D* ∈ [0.02, 0.06] m), 136 rounded small pebbles at AromaF (*D* ∈ [0.02, 0.06] m), and 134 rounded small pebbles at AromaG (*D* ∈ [0.02, 0.06] m). The 40 individual ^10^Be concentrations correspond to 15 clasts from AromaA, 20 from AromaC and five from AromaD (clast sizes are indicated in Supplementary Table S1 and in the same range as indicated above).

## Results

### Stochastic and size-dependent components of the gravel transit rates

Figure 1b shows that the mean ^10^Be concentrations below the first station AromaA are more than the double of the ^10^Be concentration at AromaA. This increase shows that more than half of the gravel ^10^Be concentration on average was acquired during their transit in the river. The mean ^10^Be concentration at AromaG (older surface) is consistently larger than the closest sample AromaF in the incised channel, demonstrating that the mean ^10^Be concentrations of the mixed gravel is at least qualitatively consistent with the geomorphology. Assuming that the mean ^10^Be concentration found in the active stream at AromaF represents a pre-deposition concentration for AromaG, the ^10^Be concentration at AromaG suggests a deposit that is 15 ± 2.6 ka in age, coinciding with a wetter period in the Atacama^35,36^. This consistency helps confirm the representativeness of these mean ^10^Be concentrations.

The mean ^10^Be concentration increases almost linearly over 43 km between AromaA and AromaD. Then, the mean ^10^Be concentration decreases and is nearly the same at the two following stations AromaE and AromaF. The difference between AromaD, AromaE and AromaF is ~30% whereas the ^10^Be concentration uncertainty is ~4%; therefore the difference is significant. The main difference between AromaD and these two stations is the size of the sampled gravel. The gravel clasts are large (10–24 cm) at AromaD, whereas they are much smaller (2–4 cm) at AromaE and AromaF. We conclude that the smaller gravel clasts were less exposed to cosmic rays on average than bigger gravel clasts. Small gravel clasts were either systematically buried at depth (smaller ^10^Be production rate), or they travelled faster on average. All along the river, we observed no grain size sorting up to several metres deep in some recent entrenchments. Thus we rule out the hypothesis that the smaller pebbles were systematically buried deeper than the larger ones. We conclude that the smaller mean ^10^Be concentration for the smaller pebbles at AromaE and AromaF means that they transited faster on average.

In addition to the downstream mean ^10^Be concentration increase, the variability in the ^10^Be concentrations in individual gravel also increases. Near the source at AromaA, the distribution of the ^10^Be concentrations at AromaA is positively skewed with a maximum that is approximately three times larger than the mean (Supplementary Fig. S2). This distribution reflects different residence times at different depths and elevations (different ^10^Be production rates) on the hillslopes. The variability in the ^10^Be concentrations increases sharply between AromaA and AromaC. The maximum ^10^Be concentration at AromaC is five times that of AromaA and the distribution at AromaC is also positively skewed with a maximum that is two times larger than the mean. At the next station, AromaD, the ^10^Be concentrations of the five individual pebbles analysed are either lower or slightly higher than the mean ^10^Be concentration of the 23 mixed pebbles, which implies that some of the 18 other pebbles in this mixture must have much higher ^10^Be concentrations. The smallest ^10^Be concentrations at AromaC and AromaD are similar to the smallest ^10^Be concentrations at AromaA, indicating that some pebbles were exposed at the river surface for a period of time less than several centuries, which is a minimum criterion for detectable ^10^Be acquisition. The large ^10^Be concentrations at AromaC correspond to pebbles with much longer exposure, and thus residence time. The large ^10^Be concentration of these slow pebbles explains the downstream increase in the station-averaged ^10^Be concentrations between AromaA and AromaD. The pebble size range sampled at AromaC is small (0.1–0.2 m), suggesting that a large variability in the ^10^Be concentrations, and therefore in the residence times, is independent of gravel size.

### Distribution of gravel residence times in the river

The data show that the distribution of residence times is controlled by a component that is inversely related to gravel size, and a stochastic component that is independent of gravel size. To go further and to establish an empirical law describing the distribution of residence times downstream, we need a model to convert the ^10^Be concentrations into residence times. This model must account for an initial grain size distribution, for the variability in the ^10^Be concentrations acquired on the hillslopes, and for the downstream evolution of these concentrations at the surface of the gravel clasts during their transit. We designed such a model based on our previous work^31^ (Supplementary Fig. S3). We start on the hillslopes with a set of gravel of various sizes including the range of sizes sampled along the river. Gravel are enriched in ^10^Be during their exhumation, either by detachment from the bedrock, or within a landslide layer, with prescribed relative probabilities adjusted to fit the ^10^Be concentrations at AromaA (see Methods). Then in the river, each pebble is transported at the surface at a constant transit rate *V* and acquires ^10^Be. Note that *V* is usually called “virtual velocity” in short-term tracer gravel studies^37^ as it averages periods of movement and periods of rest. We impose a constant *V* for each pebble because their ^10^Be concentration is essentially a record of the time spent by gravel near the surface between two locations, regardless of the details of the gravel transit and rest periods.

To account for the stochastic component of *V*, we take this value from a prescribed distribution at the beginning of its transit in the river. To account for the size-dependent component of *V*, we multiply the value taken by a function *f*(*D*) that is inversely dependent on gravel size *D*. We use an empirical law based on a compilation of short-term gravel dispersion in rivers with different climatic contexts^37^, *f*(*D*) = (1 − *log*(*D*/*D*_*o*_))^*γ*^, where *γ* = 1.35 (±0.075) and *D*_*o*_ is the median diameter in the original publication^38^. The weak dependence of *f* on *D* for *D* < *D*_*o*_ is thought to result from the higher probability for small pebbles to be trapped by larger stones on the river bed^38^. For larger gravel, *f* decreases faster than linearly with *D*. Although we did not measure the grain size in the field, we fix *D*_*o*_ = 0.14 m, which is the median gravel size that is also used in the hillslopes model. This size is bracketed by small gravel and larger cobbles observed in the river.

In the model, we successively run and record the displacement of 1500 gravel clasts of various sizes up to the river outlet and we trace the ^10^Be concentration evolution at the surface and within clasts. Once all clasts have reached the outlet, we select clasts that resided between 1 km downstream and upstream a studied river station. Their ^10^Be concentration distribution is compared to the distribution of the measured ^10^Be concentrations at this river station. The compared ^10^Be concentration corresponds either to a point at the surface of the cobbles (*D* > 0.06 m) or to the bulk ^10^Be concentration, just like the data. With regards to the cobbles, the ^10^Be concentration at their top is larger than at their bottom on the hillslopes. These two points are then traced during river transport and their ^10^Be concentration evolves by assuming that the clast rolls and settles in a random position at each step^31^. The ^10^Be concentration is randomly chosen between these two values, similarly to in the field where we took a piece of the large cobbles without knowing their initial positions.

We successively test three distributions of river virtual velocity *V* with different types of skewness, i.e. Gaussian, exponential and truncated Pareto distribution. We evaluate their consistency with the data by minimizing the misfit between the measured and predicted mean ^10^Be concentrations, as well as the misfit between the measured and predicted distributions of the ^10^Be concentrations at AromaC (see Methods).

**Figure 2:**
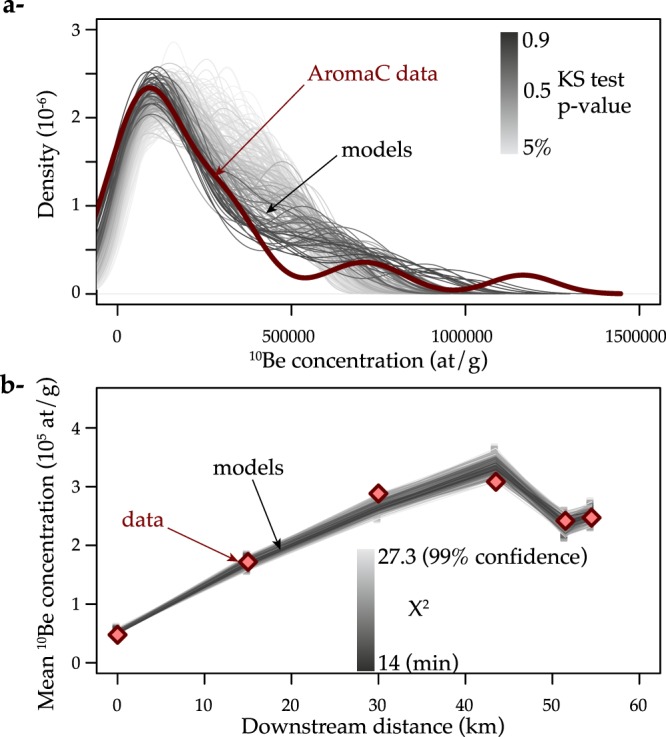
Results of model fit considering constant transport rates drawn from a truncated Pareto distribution $({\rm{pdf}}(V)=\frac{\alpha +1}{{1000}^{\alpha }-{V}_{Min}^{\alpha }}{V}^{\alpha })$ and multiplied by (1 − *log*(*D*/0.14))^1.35^ as suggested by short-term gravel tracer studies^37^. (**a**) Distribution of AromaC ^10^Be concentrations and acceptable models for different values of *V*_*oMin*_ and *α*. (**b**) Station-averaged ^10^Be concentrations and acceptable models. The acceptable models are those that satisfy the Komosgorov and Smirnov (KS) test with 95% confidence (p-value > 5%) for AromaC and also correspond to 99% confidence based on *χ*^2^ minimisation (see Methods). The acceptable models correspond to *V*_*Min*_ ∈ [0.4, 0.93] m a^−1^ and *α* ∈ [−1.94, −1.04]. The best fit model (lowest *χ*^2^ and AromaC p-value > 5%) corresponds to *V*_*Min*_ = 0.55 m a^−1^ and *α* = −1.2 (Supplementary Fig. S4).

We find that the model fits the data if the imposed distribution of the *V* values is a truncated Pareto distribution (Fig. 2 and Supplementary Fig. S4), with a power law tail on the form ${\rm{pdf}}(V)=\frac{\alpha +1}{{V}_{Max}^{\alpha +1}-{V}_{Min}^{\alpha +1}}{V}^{\alpha }$, with *α* ∈ [−1.94, −1.04], and *V*_*Min*_ ∈ [0.4, 0.93] m a^−1^ for *D*_*o*_ = 0.14 m gravel (*V*_*Max*_ is fixed to 1 km a^−1^ and larger values do not change the results). In fact, a Gaussian distribution of *V* cannot reproduce the skewness of the ^10^Be concentration distribution at AromaC and the mean ^10^Be concentrations at the same time (Supplementary Fig. S5a). Exponential distributions of *V* result in ^10^Be concentration distributions that meet the requirements of the statistical tests (see Methods), but which systematically underestimate the frequency of small ^10^Be concentrations at AromaC (Supplementary Fig. S5b–d). Pareto distributions of *V* best reproduce the data (Fig. 2). The acceptable models fit the skewness of the ^10^Be concentrations well at AromaC and the other mean ^10^Be concentrations. This simple model fits the decreases in the ^10^Be concentration well for the smaller gravel at AromaE and AromaF. This decrease is due to the modelled size-dependent *V* relationship. Despite the large imposed variations in *V* for a given *D*, smaller pebbles go faster on average and thus their mean ^10^Be concentration is smaller than the coarser pebbles sampled at AromaD. Given the strong assumption that the short-term empirical law could be extrapolated over millennia, the consistency between this model and the data is remarkable. Furthermore, we tested different values of exponent *γ* of the velocity-size relationship and we found that the best fit is obtained with the same exponent 1.35 as determined from short-term tracers (Supplementary Fig. S6).

The addition of the possibility that pebbles are temporarily buried in river sediment with an equal depth probability slightly improves the fit for the AromaC ^10^Be concentrations but does not change the range of acceptable exponents in the Pareto distributions (Supplementary Fig. S7). When a gravel resides at depth, the ^10^Be production rate is lower and thus its ^10^Be concentration evolves more slowly. The longer gravel residence at depth is compensated by a faster velocity when it moves to fit the observed ^10^Be concentrations, leading to similar ranges of inferred *V* distributions. Testing a smaller *D*_*o*_ value (*D*_*o*_ = 0.08 m) does not change our conclusions (Supplementary Fig. S8).

Last, we tested the effect of gravel size reduction via abrasion by progressively stripping off the surface of the gravel so that their size decreases exponentially with the travel distance^39,40^ (Supplementary Fig. S9). In the model, the attrition of a pebble accelerates its transport and slightly decreases its surface ^10^Be concentration, but these modifications are negligible compared to the variability in *V* required to fit the ^10^Be concentrations. As a result, gravel abrasion does not modify our outcomes. The splitting of a cobble into smaller pieces (*D* < 10 cm) is not taken into account by the model. The addition of these pieces to the small gravel at AromaE and AromaF would increase their mean ^10^Be concentrations. This would decrease the inferred difference in *V* values between small and large gravel. Thus, the size-dependent *V* evidenced from our data is robust.

**Figure 3:**
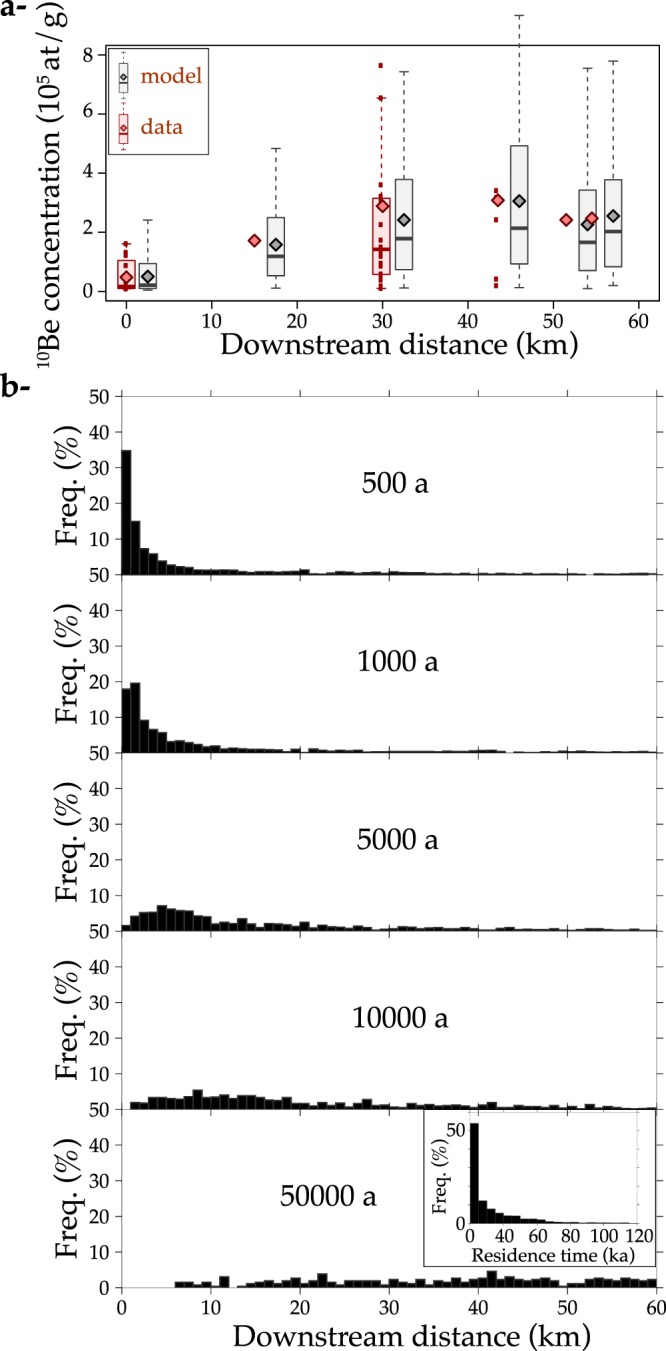
Predictions of the best-fit model shown in Fig. 2 (pdf(*V*) = 0.23*V*^−1.2^). (**a**) Comparison of the predicted ^10^Be concentration distributions with data at the different river stations. (**b**) Downstream spreading of an initial population of 1500 gravel clasts in the range *D* ∈ [0.02, 0.72] m. Part of the gravel (including large pieces) reaches the river outlet after a couple of decades, whereas other gravel clasts are still in the river after 50 ka. The inset graph shows the resulting distribution of residence times *τ* (∝*τ*^−1^) in this 56 km-long river. For one specific gravel size, the predicted distribution of residence time *τ* is pdf(*τ*) = pdf(*V*)*dV*/*dτ* ∝ *τ*^−*α*−2^ ∝ *τ*^−0.8^ with *α* = −1.2.

Once the best-fit distribution of the virtual velocities has been established, we can use it to predict the downstream spreading of an initial mixture of 1500 gravel clasts with the same size range as at AromaA. Figure 3 shows snapshots of the gravel plume at different times. A small fraction of the gravel clasts reaches the river outlet in less than several centuries, but it then takes several tens of thousands of years to evacuate all of the gravel clasts. The resulting distribution of gravel residence times between the gneissic source and the river outlet has a power law tail (∝*τ*^−1^), indicating that a significant fraction of the gravel (6.5%) reaches the river outlet after a very long residence time (>100 ka).

## Discussion

By documenting, for the first time, the distribution of the ^10^Be concentrations from a source to the outlet of a river, our data show that it is possible to acquire a large variability in terms of the gravel ^10^Be concentrations during their transit within a 56 km-long river. These data complement recent high ^21^Ne concentrations found in gravel from long rivers in the Great Plains (USA) which is interpreted as evidence of long residence times^8^. In the Aroma River, the ^10^Be concentrations correspond to highly variable transit rates (*V* > 0.4 m a^−1^ for 0.14 m gravel) that are larger than the mean transit rates obtained for sand in the Mojave Desert piedmont (~0.16 m a^−1^)^20,41^, probably because the transport is laterally constrained in the Aroma River case. In order to explain part of the differences in the ^10^Be concentration observed in some cases between sand and pebbles worldwide, it has been suggested that gravel may not accumulate detectable ^10^Be concentrations during their transport in channelized rivers^30,42–47^. The observed downstream increase in the ^10^Be concentration in the Aroma River demonstrates this possibility for arid environments. Such ^10^Be concentrations would correspond to a small inheritance that is unlikely to bias the ages of the Mio-Plio-Pleistocene alluvial surfaces in this region^32,48,49^. For younger deposits, however, a positively skewed distribution of pre-deposition ^10^Be concentrations can be expected to strongly contribute to the final distribution of ^10^Be concentrations used to calculate the exposure ages. These different ^10^Be concentrations can explain different ages for the same deposit. For example, high and variable pre-deposition ^10^Be concentrations were found in gravel and boulders of post-20 ka alluvial fans at the front of the Andes in Argentina^50^. Although the hillslopes and river contributions to this variability could not be determined in that case, our results show that the transport itself in the river can explain the high ^10^Be concentrations (outliers) in gravel deposited on active alluvial fans.

The variability in gravel transit rates is probably linked to different temporary storage periods and transport lengths during erosive floods. If the storage was deep (>>1 m), the increase in the ^10^Be concentration would be undetectable. The observed downstream ^10^Be concentration increase shows that the storage of highly concentrated gravel occurred mainly in the subsurface, and thus probably in bars and lateral deposits. These gravel clasts are then either reentrained or recycled by the active streams. Recycling has been identified as a major issue for interpreting the grain size and mineralogy of basins in terms of tectonics and climate, but it is difficult to prove and quantify^7^. Sediment recycling in an alluvial fan was recently shown to control the downstream gravel grain size variations in alluvial fans found in an arid Andean piedmont in Argentina^51^. Our data confirm that sediment storage and recycling occur even in this relatively short and confined river. Nevertheless, the proportion of recycled gravel in the gravel flux is difficult to estimate. In a first attempt, we can define the proportion of recycled gravel as the proportion of gravel with residence times longer than the residence time predicted in the simple case of steady gravel flux^8^. By multiplying the Aroma catchment area by a denudation rate of 10 m/Ma, which is characteristic for this area^52,53^, we estimate a sediment flux of 10^4^ m^3^ a^−1^. The volume of the transported sediment in the river is estimated by multiplying the river length of 56 km by the mean valley width of ~100 m and by a conservative sediment mixing depth of ~10 m. Dividing the volume of transported sediment by this flux, we obtain a conservative maximum estimate of the mean gravel residence time in the river of ~5600 a. In the best-fit scenario illustrated by Fig. 3, 58% of the gravel have residence times longer than 5600 a. This is a crude estimate because gravel size should be taken into account, yet it allows us to conclude that long storage times and the recycling of sediment are processes that control the spreading of a gravel plume over long timescales in the Aroma River^13^.

Despite the stochasticity of gravel transit rates, our results demonstrate that small pebbles travel faster, on average, than larger ones over millennial timespans in this river. Our data fits the size-velocity relationship *f*(*D*) that was established previously from step lengths of gravel during individual floods^37^. This relationship arises from the size-dependent entrainment probability and transport distances^37^. It is likely that in our model, *f*(*D*) also reflects this behaviour of the gravel during floods^54^, which means that the size-dependent entrainment probability and transport distances still hold over the full range of floods over millennia. Our results thus suggest that the form of *f*(*D*) is universal.

This size-velocity relationship predicts that big gravel would have a larger probability to be buried in a subsiding basin, and would therefore imply the downstream fining of gravel. The downstream fining of gravel is observed in most basins and has been modelled experimentally^9,55^. From these observations, a simplified self-similar solution for the long-term substrate grain-size distribution has been proposed^56^ and successfully used to reconstruct subsidence rates and input flux histories in some examples^57,58^. A recent study has shown that this model is improved by including the lateral inputs of recycled sediments^51^. Our results confirm that lateral storage and recycling contributes to the downstream grain size evolution. Combined with an erosion-sedimentation mass balance equation^59^, the distribution of the size-dependent transit rates established in our study could be used to predict the river bed elevation and the fluxes of different gravel sizes according to grain size distributions supplied by lateral hillslopes. Such a modelling approach may complement models based on self-similarity hypothesis.

Finally, recent studies have debated on the diffusive nature of sediment transport. The diffusion equation is used to predict the long-term fluvial evolution in response to tectonic or climatic perturbations^9^. Small-scale river experiments^60^ have suggested that diffusion explains the buffering of sediment inputs. Numerical models have used diffusion theory to predict the frequency of sediment pulses that could be recorded in basins^4,9,10^. Yet, this theory has not been confirmed over the long term^61^. For the first time, our data provide some support for diffusion by evidencing the downstream spreading of a gravel plume (Fig. 3).

Yet, the parallel with diffusion is not perfect. The diffusion theory applied to an initial punctual population of gravel would predict that the distribution of residence times (“first-time passage” in diffusion theory^62^) is a Levy distribution. This distribution is very positively skewed and has a power law tail with a well-defined exponent −1.5. In our case, the inferred distribution of residence times in the best fit model has a power law tail with a larger exponent ~−1, i.e. a heavier tail. This value means that there is a higher probability of obtaining very large residence times. As a result, a larger proportion of slow gravel can be stored upstream for a longer time than predicted by diffusion. Consequently, our data suggest an anomalous diffusion.

This result echoes recent findings based on short-term tracer gravel in rivers and small-scale experiments that identified anomalous diffusion^11,13,14,16,17,63–66^. There is some consensus that anomalous diffusion arises from heavy-tailed distributions of sediment storage periods^16^. Such a distribution may be consistent with our inferred heavy-tailed distribution of residence times. Indeed, our data are explained by a simple model with two components, a size-dependent term and a power law distribution of mean transit rates. The first size-dependent term comes from empirical observations worldwide of gravel transport during floods. We suggest that this term has the same meaning over long timescales in Aroma, i.e. that it describes a size-selective movement of pebbles during floods in the river. Conversely, we suggest that the second component, the power law *V* distribution, arises from variable periods of gravel storage. In the case of Aroma River, this distribution is probably controlled by the recycling of sediment stored in the alluvial cover of the floodplain, possibly in bars and lateral deposits, which occurs over much longer periods of time than those covered by short-term tracer gravel surveys.

The origin of the anomalous diffusion evidenced in laboratory experiments on aggrading braided rivers is still unclear^12^. In the Aroma River, the lateral long storage of particles and their posterior recycling provide an explanation that may also hold true in laboratory experiments.

Anomalous diffusion could change our view of the landscape dynamics. When a large amount of coarse material is occasionally generated in a catchment by a landslide, this pulse of sediment will reach towns located downstream at different times and spread during different periods depending on whether diffusion or anomalous diffusion dominates. Because increased gravel flux can cause river bed aggradation and flooding^67^, the identification of anomalous diffusion may have implications for flooding hazard modelling at centennial timescales or even longer. Anomalous diffusion can also influence the concavity of rivers and thus the dynamics of the whole landscape. Although most models have considered that river concavity is linked to the physics of bedrock detachment and to the distribution of floods^2^, anomalous diffusion of the gravel cover is predicted to control the concavity and alluvial thickness distribution along mixed bedrock-alluvial rivers^68^. In sedimentary basins, anomalous diffusion may generate power law distributions of non-deposition or non-erosion periods. In that case, it is predicted that the reconstruction of the sedimentation and erosion rate histories over geological timescales are strongly biased^69–71^. A more in-depth long-term evaluation of anomalous diffusion as well as further investigations into its consequences on understanding the dynamics of mountain relief are needed. Meanwhile, our study provides new guidelines to document the gravel dynamics in other contexts, and new indicators for modelling the long-term sediment transport dynamics from the point of view of individual gravel clasts.

## Methods

### ^10^Be concentrations

We collected samples throughout the month of May in 2010 (Supplementary Fig. S1). Then, the samples were crushed with a jaw crusher and sieved to keep the granulometric fraction between 0.5 and 1 mm. This work was carried out at GET (Géosciences Environnement Toulouse). Afterward, the chemical treatment of the samples was performed at the LN2C (Laboratoire National des Nucléides Cosmogéniques), CEREGE, Aix-en-Provence. First, magnetic minerals were separated from the bulk by a magnetic separator (Frantz LB-1). Then, the non-magnetic fractions underwent a series of acid attacks with a mixture of concentrated hydrochloric and hexafluorosilisic acids to remove all non-quartz minerals. When the quartz was extracted, meteoric ^10^Be was removed by three partial dissolutions with concentrated hydrofluoric acid. The decontaminated quartz was totally dissolved with concentrated hydrofluoric acid after adding of 100 *μ*L of an home-made ^9^Be carrier solution^72^ ([^9^Be] = 3025 ± 9 *μ*g/g). The resulting solutions were evaporated until dryness and the samples were recovered with hydrochloric acid. Then, the samples were precipitated with concentrated ammonia before a successive separation through an anion exchange column (Dowex 1X8) to remove the iron and a cation exchange column (Dowex 50WX8) to discard the Boron and recover the Be^73^. Finally, the eluted Be was precipitated to Be(OH)_2_ with concentrated ammonia and oxidized to BeO. After target preparation by mixing Niobium powder with the BeO oxide, the ^10^Be/^9^Be ratios were measured by Accelerator Mass Spectrometry (AMS) at the French National AMS Facility ASTER of CEREGE in Aix-en-Provence^74^. The measured ^10^Be/^9^Be ratios in 2012 and 2013 were directly calibrated against the National Institute of Standards and Technology Standard Reference Material 4325 NIST with an assigned value^75^ of (2.79 ± 0.03).10^−11^. The measured ^10^Be/^9^Be ratios in 2015 were calibrated against a house standard STD-11 with an assigned value^76^ of (1.191 ± 0.013).10^−11^. The analytical 1 *σ* uncertainties include uncertainties in the AMS counting statistics, the uncertainty in the standard ^10^Be/^9^Be, an external AMS error of 0.5%^74^, and a chemical blank correction. A ^10^Be half-life of (1.387 ± 0.01).10^6^ years was used^77,78^.

### Hillslope gravel model

Because the ^10^Be production rate depends on the elevation, which varies downstream, we need a model to convert the ^10^Be concentrations into transport velocities. The model^31^ calculates the ^10^Be concentration *N* (at g^−1^) at the surface of a gravel during its transport along an elevation profile (Supplementary Fig. S3). *N* is a solution of

1 $$\frac{dN}{dt}=P-\lambda N$$

where $P={\sum }_{i=1}^{3}\frac{{P}_{i}}{\frac{\rho \varepsilon }{{\mu }_{i}}+\lambda }{e}^{\frac{-\rho z}{{\mu }_{i}}}$ is the ^10^Be production rate (at g^−1^ a^−1^), *i* stands for neutrons, fast and slow muons, *P*_*i*_ are the surface ^10^Be production rates associated with each particle^79^ scaled for latitude and elevation^80^ using a sea-level/high-latitude production rate^81^ of 4.0 at g^−1^ a^−1^, *μ*_*i*_ = 150, 1500 and 5400 g cm^−3^ are the corresponding attenuation coefficients^79^, *ρ* = 2.5 g cm^−3^ is the rock density, *ε* is the surface erosion rate, *λ* = 4.99 10^−7^ a^−1^ is the radioactive decay^77^, and *z* is the depth.

The elevation profile is divided into a hillslope part (gneiss elevations between 2919 and 2069 m) and the elevation profile of the main river of the aroma catchment (2069 to 1072 m). On the hillslope part, a gravel is randomly detached either from the bedrock surface or in a landslide to account for both phenomena observed on the Aroma hillslopes. If detached from the bedrock, the ^10^Be concentration of the gravel corresponds to the steady-state long-term balance between the ^10^Be lost by erosion and radioactive decay and production^31^ at a randomly selected elevation between 2069 and 2919 m. If the gravel is in a landslide, the landslide thickness *H*, the pebble elevation and its depth in this landslide are drawn according to a uniform probability distribution between 0 and *H*. For a landslide-derived gravel, the probability *p* to belong to a landslide with a thickness between *H* and *H* + *dH* is the relative volume of sediment produced by all landslides of that size over the long-term, or

2 $$p=\frac{AH\,{\rm{pdf}}(H)dH}{{\int }_{{H}_{min}}^{{H}_{max}}AH\,{\rm{pdf}}(H)dH}$$

where pdf(*H*) is the probability density function of landslides with a thickness *H*, *H*_*min*_ and *H*_*max*_ are the minimum and maximum landslide thicknesses and *A* is the landslide area. pdf(*H*) is determined by applying the change-of-variable technique to the frequency-area pdf of the landslides^82^

3 $${\rm{pdf}}(A)\propto {A}^{-\beta }$$

where *A* is the landslide area and *β* is a scaling exponent, and using a thickness-area relationship^82^:

4 $$H\propto {A}^{0.5}$$

This leads to

5 $$p=\frac{5-2\beta }{{H}_{max}^{5-2\beta }-{H}_{min}^{5-2\beta }}{H}^{4-2\beta }dH$$

We estimate *H*_*max*_ = 10 m from the observations. *β* and *H*_*min*_ are unknown and are estimated by fitting the AromaA gravel ^10^Be concentrations (see below). The few data available for the gravel size distribution on hillslopes worldwide^83^ suggest a log-normal distribution^39^ in some cases. We use a log-normal distribution of log-mean 0 and log-*σ* 1.5 and only retain *D* values between 0.02 and 0.72 m corresponding to the range of the sampled gravel. The choice of *σ* = 1.5 ensures 95% of gravel within that range. The mean *D* is 0.17 m and the median is 0.14 m. Last, we observed that our conclusions are not sensitive to the initial grain size distribution (Supplementary Fig. S10). In a landslide, the gravel ^10^Be concentration is the sum of a steady-state ^10^Be concentration representing the long-term balance between gain and loss at the gravel depth and a ^10^Be concentration corresponding to the gravel residence time in the landslide layer^31^. The residence time is *H*/*ε* where *ε* is the long-term mean erosion rate due to landslides.

For the hillslope part of the model, we find that 30% of the clasts detached from the bedrock at a time-average rate of 0.17 mm a^−1^ and 70% of the clasts exported by landslides at a time-averaged erosion rate of 1 mm a^−1^ fit the two peaks in the ^10^Be concentration distribution for AromaA (Supplementary Fig. S11). These erosion rates are high in the Chilean context but consistent with the active shallow seismicity of this area^84^ and other ^10^Be pebble-derived erosion rates in northern Chile^53,85,86^. The landslide parameters *β* and *H*_*min*_ are then estimated by fitting the AromaA distribution of the ^10^Be concentrations with the modelled ^10^Be concentration distributions. We generate 2500 models (1500 gravel per model) by varying *β* between 1.1 and 2.9 (range of the estimated values worldwide^87^) and *H*_*min*_ between 0.1 and 1.5 m. We accept models for which the p-value of the Komolgorov-Smirnov (KS) test is larger than 5% (therefore we only accept a 5% chance of erroneously rejecting models that belong to the same ^10^Be concentration distribution as the data). The corresponding minimum and maximum values of *β* and *H*_*min*_ determine the 95% confidence intervals for these parameters. At this confidence level, it turns out that we cannot reject any of the tested *β* and *H*_*min*_ values according to the KS test (all p-values are >5% and are similar - Supplementary Fig. S11). Thus, we retain the values *H*_*min*_ = 0.7 m and *β* = 2.3, an intermediate exponent also found in southern Peru^87^. Other values do not affect our results because they produce a similar distribution of the ^10^Be concentrations at AromaA (Supplementary Fig. S11a).

### River parameters fitting

The fitting procedure for the river data assumes a time-constant distribution of the ^10^Be concentrations on the hillslopes. This assumption is justified by the undistinguishable distributions of the gravel ^10^Be concentrations measured in the Veladera River (southern Peru) and in its adjacent ~16 ka old terrace^87^. Assuming a Pareto distribution of *V* of the form given in the main text, *V*_*Min*_ and *α* are determined by the following procedure. Using the best-fit *β* and *H*_*min*_ parameters, we generate models by varying *α* (∈[−2.5, −0.5]) and *V*_*Min*_ (∈[0.3, 1.5] m a^−1^) and calculate the p-value of the KS test. For each model, we also calculate

6 $${\chi }^{2}=\mathop{\sum }\limits_{i=1}^{6}\,{(\frac{{y}_{i}-{x}_{i}}{{\sigma }_{i}})}^{2}$$

where *y*_*i*_ are the modelled station-averaged ^10^Be concentrations, and *x*_*i*_ and *σ*_*i*_ are the corresponding measured station-averaged ^10^Be concentrations and uncertainties, respectively. Both tests are carried out by selecting the simulated gravel at each river station belonging to the same size interval as the gravel collected in the field. The acceptable models finally satisfy both *χ*^2^ < *χ*_*min*_^2^ + 13.3 (13.3 is a tabulated Δ*χ*^2^ cut-off value corresponding to a 99% confidence level for four degrees of freedom) and the KS test on AromaC (p-value > 0.05). The corresponding minimum and maximum values of *V*_*Min*_ and *α* define the 99% confidence intervals. The best-fit model is the model with the highest p-value for the KS test applied to AromaC among the models for which *χ*^2^ < *χ*_*min*_^2^ + 13.3 (Supplementary Fig. S4). The same procedure is applied to test other distribution of *V* (Supplementary Fig. S5).

## Supplementary information

Supplementary Figures

Table S1

model codes

## Data Availability

An archive called COSMOBOULDER.zip contains all the codes used in this study and README file explaining how to use them.
