# Supplementary material for: Long-term dispersion of river gravel in a canyon in the Atacama Desert, Central Andes, deduced from their 10Be concentrations
Source: Sci Rep. 2019 Nov 28;9:17763. doi: 10.1038/s41598-019-53806-x (PMC6882893; doi:10.1038/s41598-019-53806-x)
Supplement: Supplementary file 1 — Supplementary Figures [file 41598_2019_53806_MOESM1_ESM.pdf]

Supplementary Material of "Long-term dispersion of river gravel in a canyon in the Atacama Desert, Central Andes, deduced from their 10Be concentrations"

Sébastien Carretier[1,*], Vincent Regard[1], Laëtitia Leanni[2], and Marcelo Farías[3]

[1]GET, Université de Toulouse, CNRS, IRD, UPS, (Toulouse), France
[2]Aix Marseille Univ, CNRS, IRD, Coll France, CEREGE, Aix-en-Provence, France
[3]Department of Geology, FCFM, University of Chile, (Santiago), Chile

ABSTRACT

Intense storms or earthquakes in mountains can supply large amounts of gravel to rivers. Gravel clasts then travel at different rates, with periods of storage and periods of displacement leading to their downstream dispersion over millennia. The rate of this dispersion controls the long-term downcutting rate in mountainous rivers as well as the grain-size signature of climate and tectonic variations in sedimentary basins. Yet, the millennial dispersion rates of gravel are poorly known. Here, we use ^{10}Be concentrations measured in individual pebbles from a localized source along a 56 km-long canyon in the Central Andes to document the distribution of long-term gravel transit rates. We show that an inverse grain-size velocity relationship previously established from short-term tracer gravel in different rivers worldwide can be extrapolated to the long-term transit rates in the Aroma River, suggesting some universality of this relationship. Gravel are also dispersed by large differences in the mean transport rates independent of gravel size, highlighting that some gravel rest at the river surface over tens of thousands of years. These different transport rates imply a strong spreading of the gravel plumes, providing direct proof for the long-term river buffering of sediment signals between mountainous sources and sedimentary basins. The inferred distribution of residence times suggests the first evidence of anomalous diffusion in gravel transport over long timespans.

References

1. Church, M. & Hassan, M. Size and distance of travel of unconstrained clasts on a streambed. *Wat. Resour. Res.* **28(1)**, 299–303 (1992).

2. Attal, M. & Lave, J. Changes of bedload characteristics along the Marsyandi River (central Nepal): Implications for understanding hillslope sediment supply, sediment load evolution along fluvial networks, and denudation in active orogenic belts. In Willett, S., Hovius, N., Brandon, M. & Fisher, D. (eds.) *Tectonics, Climate, and Landscape Evolution*, vol. 398, 143–171, DOI: 10.1130/2006.2398(09) (Geological Society of America Special Paper, 2006).

3. Attal, M. & Lave, J. Pebble abrasion during fluvial transport: experimental results and implications for the evolution of the sediment load along rivers. *J. Geophys. Res. Earth Surf.* **114**, F04023, DOI: 10.1029/2009JF001328 (2009).

Supplementary Material

[see SupplementaryDataTable1.xls file]

Supplementary Tab. S 1. Results of AMS measurements, sample location and size.

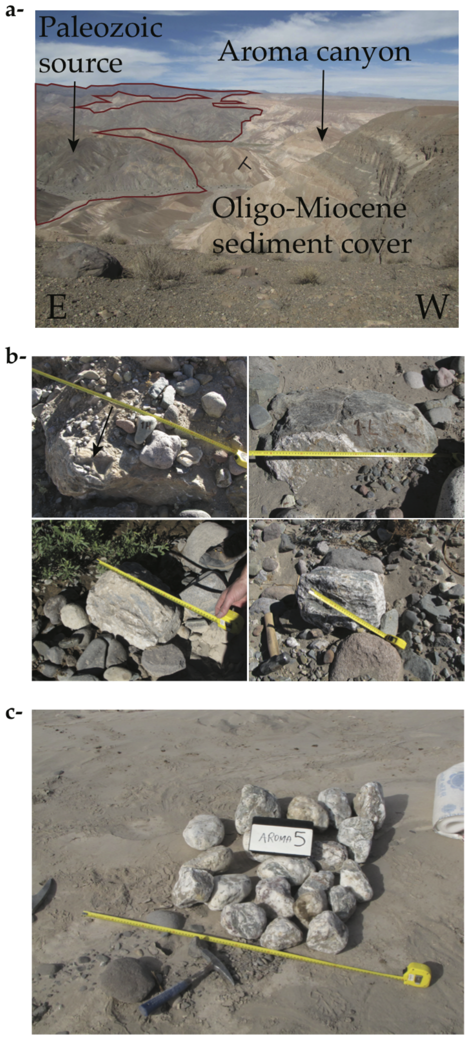

Supplementary Fig. S 1. Field photos. (**a-**) View towards the south at the start of the Aroma Canyon just downstream from the Palaeozoic gneiss rocks sampled downstream. Note that these rocks are exposed within an erosion window. Oligo-Miocene sediments were covering the Palaeozoic rocks up to the opening of the erosion window associated with the canyon incision. (**b-**) Some examples of sampled cobbles at AromaA. (**c-**) The pebbles sampled at AromaC.

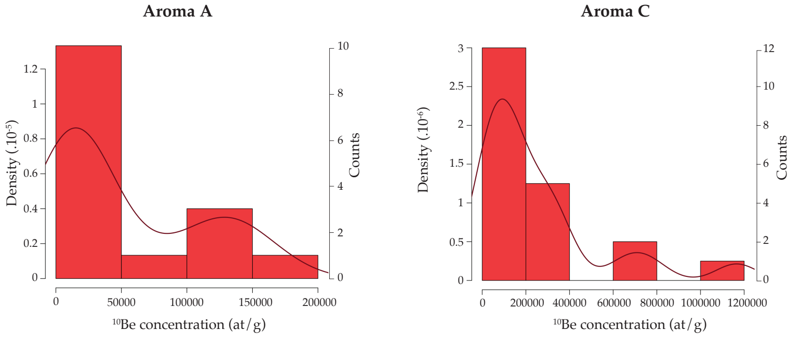

Supplementary Fig. S 2. Histogram and density plot of ^{10}Be concentrations at AromaA and AromaC.

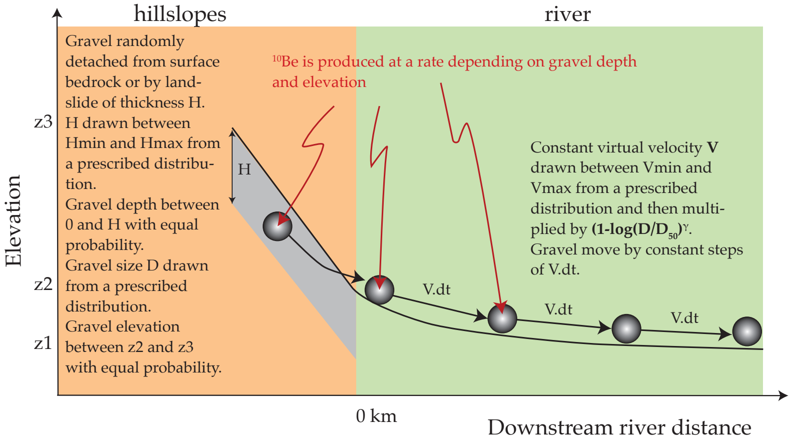

Supplementary Fig. S 3. Schematic drawing (not to scale) of the ^{10}Be concentration model for one gravel. In the river gravel are moved by time steps dt=20 a.

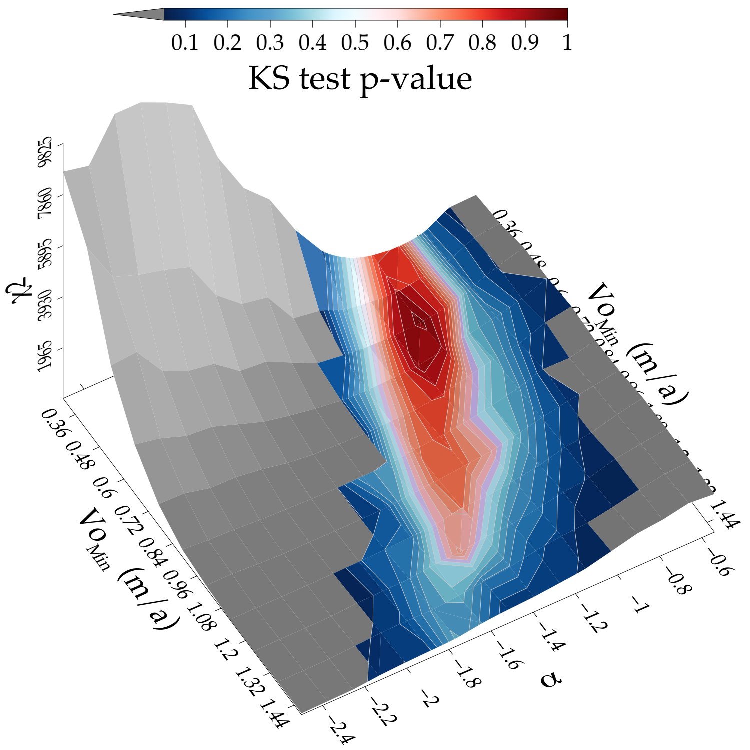

Supplementary Fig. S 4. Results of the model fit considering a mean transport velocity V is a function of gravel diameter[1] D ($f(D) = (1 - log(D/0.14))^{1.35}$) and varies from one gravel to another following a Pareto distribution $\mathrm{pdf}(V) = \frac{\alpha+1}{1000^{\alpha+1} - V_{Min}^{\alpha+1}} V^{\alpha}$ (Figure 2). The p-values $> 5\%$ for the KS test overlying the χ^2 surface for 10000 tested models varying V_{Min} and α. The best fit model (lowest χ^2 and Aroma 5 p-value>5%) corresponds to $V_{Min} = 0.55$ m a^{-1} and $\alpha = $ -1.2.

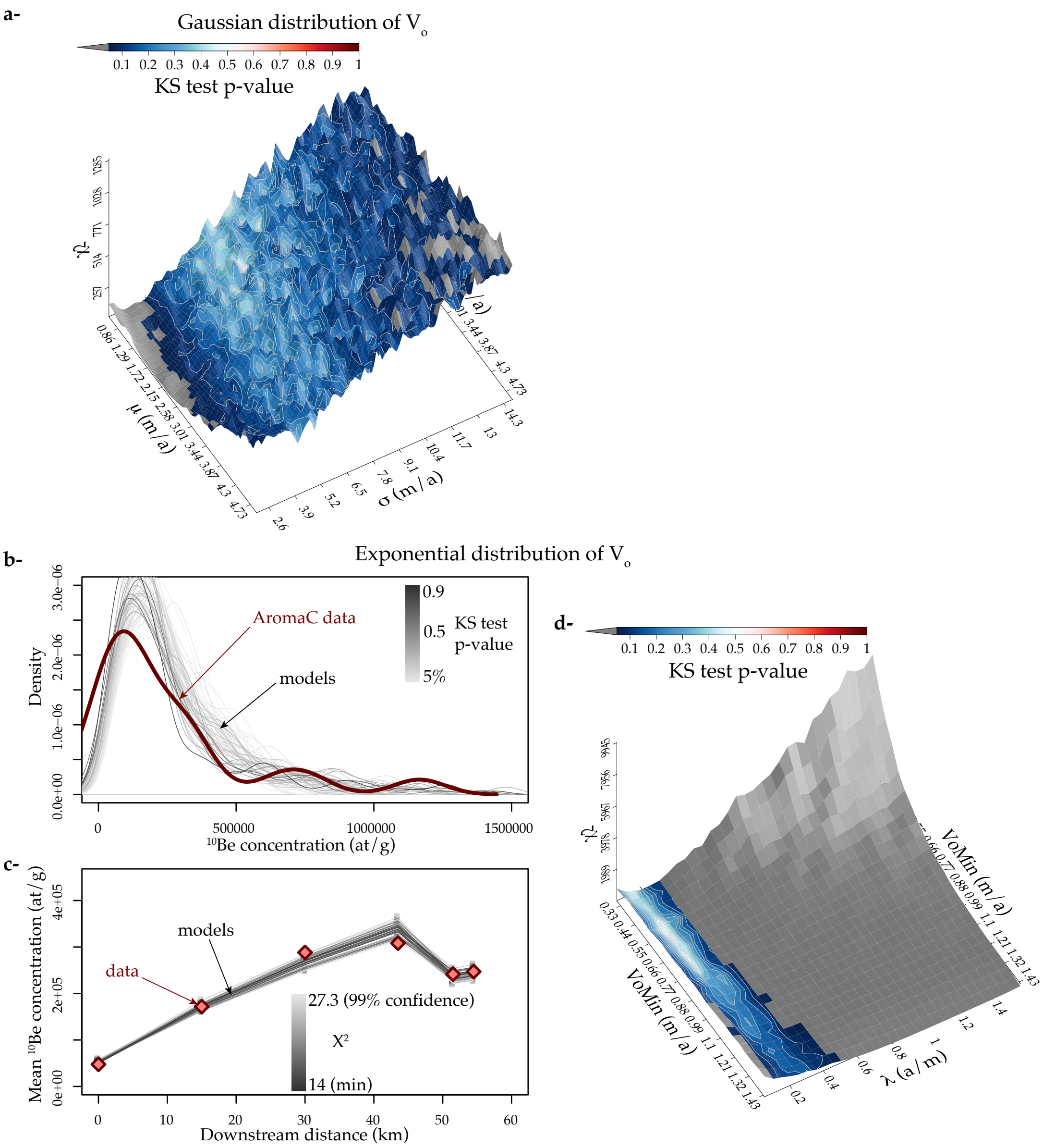

Supplementary Fig. S 5. Attempt to fit data with other distributions of V. **a-** The imposed distribution of V is a normal distribution of V truncated between V_{Min} = 0.55 m a^{-1} (best value to fit the highest ^{10}Be concentration at AromaC) and V_{Max} = 1000 m a^{-1} where μ is the mean of the normal distribution and σ is its standard deviation. The space domain of models with a p-value for the KS test applied to AromaC > 0.05 is draped above the χ^2 surface. There is no overlap between this domain and low χ^2 values ($\chi^2 < \chi^2_{min} + 13.3$). Thus, a normal distribution V_o cannot be used to fit the distribution (reflected by the KS p-values) and the average ^{10}Be concentrations (χ^2). **b-** to **d-** The imposed distribution of V_o is a truncated exponential distribution ($\frac{\lambda e^{-\lambda V}}{e^{-\lambda V_{Min}} - e^{-\lambda 1000}}$). **b-** and **c-** Acceptable models satisfying the statistical tests. **d-** p-values of the KS test for AromaC overlying the χ^2 surface. The p-values of the acceptable models (p-value$>$ 0.05) are lower ($<$ 0.6) than those with a Pareto distribution ($<$ 0.9) of V (Fig. **??**c) because the acceptable models systematically underestimate the frequency of small ^{10}Be concentrations (**b-**), which does not occur with a Pareto distribution of V_o (Fig. **??**a).

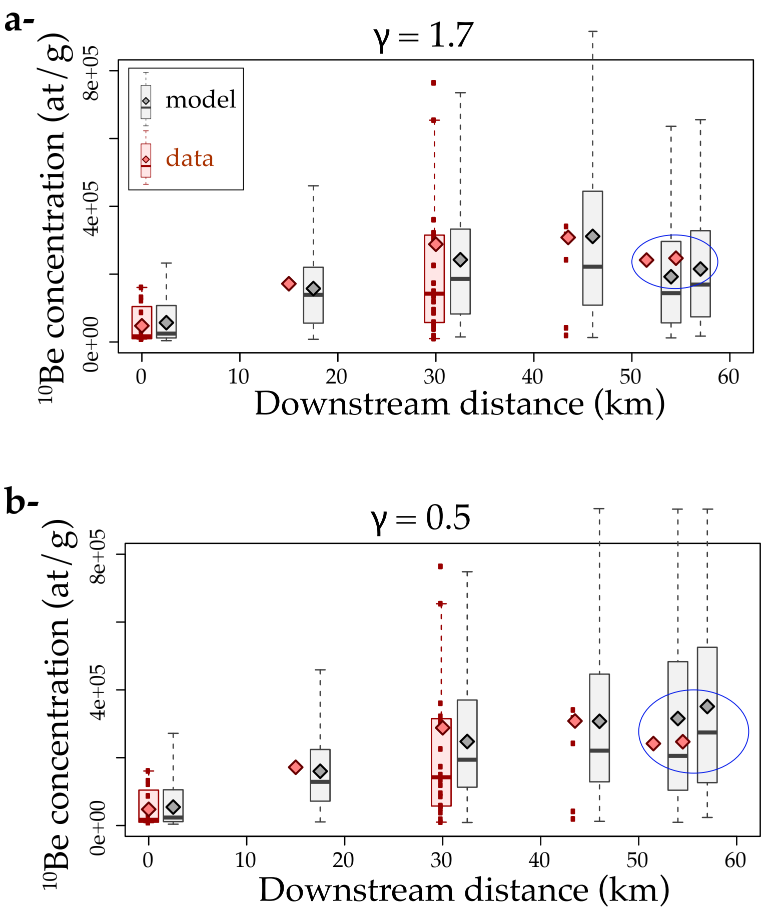

Supplementary Fig. S 6. Same as Fig. 2a but using a different exponent γ in the size-velocity relationship function of gravel size $f(D) = (1 - log(D/D_o))^{\gamma}$. The value of γ inferred from a compilation of traced gravel travel distance measurements according to gravel size gave[1] $\gamma = 1.35$. This value was used in all our experiments. By using a higher (**a-**) and lower (**b-**) value, the predicted station-averaged ^{10}Be concentrations of the two last sampling points (blue circles) are underestimated and overestimated, respectively. This difference occurs because the exponent γ influences the difference in the mean velocities between large and small gravel. As the gravel sampled at the two last sampling points are smaller than those upstream, their station-averaged ^{10}Be concentrations depends on γ. This figure shows that, over millennia, the best-fit models (Fig. 2a) are obtained by using the same exponent γ deduced from the compilation of short-term tracer pebble distances.

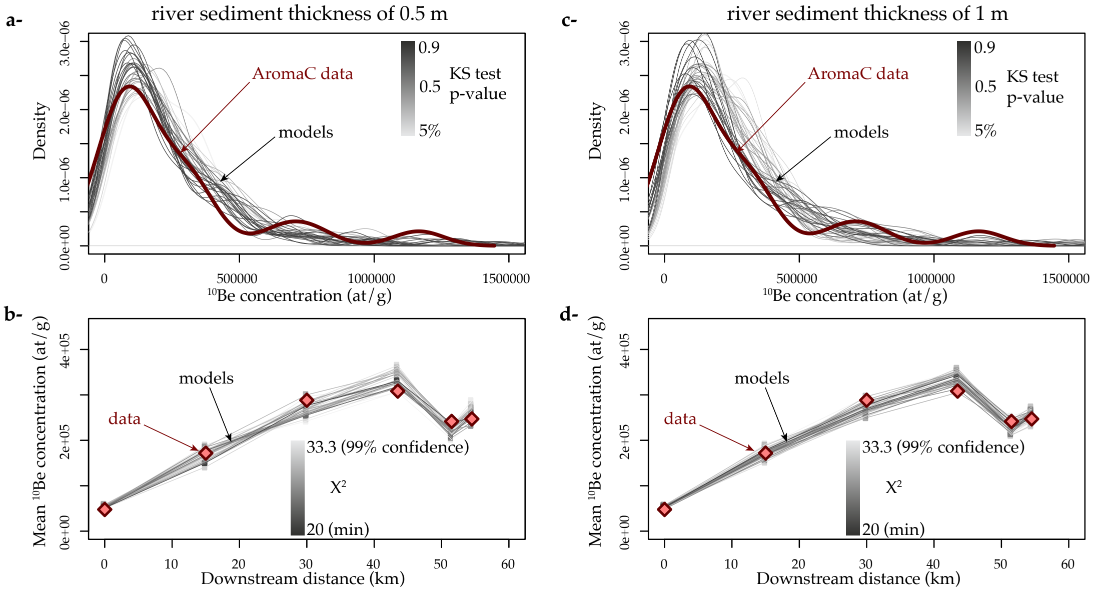

Supplementary Fig. S 7. Same as Fig. 2 but at each time step, the gravel depth is drawn with a uniform probability between depth 0 and the thickness z_{max} of a constant and homogeneous river sediment mixing layer. If the gravel top is exposed above the sediment layer, the gravel jumps to a distance $V.dt$. **a-** and **b-** z_{max} =0.5 m, V_{oMin} ∈[3.12,4.41] m a^{-1} and α ∈[-1.82,-0.78]. **c-** and **d-** z_{max} =1 m, V_{Min} ∈[1.59,2.94] m a^{-1} and α ∈[-1.86,-0.94]. V_{oMin} is larger with z_{max} =0.5 m than with z_{max} =0 m (Fig. 2) to compensate for the longer residence in the high ^{10}Be production layer near the surface. For z_{max} =1 m, V_{Min} is smaller because gravel spend a significant fraction of time at depth where the ^{10}Be production rate is much smaller. Note that in these cases where gravel spend intermittent periods in a sediment layer, the inferred V values are the minimal values of the virtual velocities (including periods of rest at depth). For $D_o = 0.14$ m, the virtual velocity is $\bar{V} = \frac{D_o}{z_{max}}V$. Thus the minimum virtual velocity $\bar{V}_{Min}$ ∈[0.87,1.23] in **a-** and **b-**, and $\bar{V}_{Min}$ ∈[0.22,0.41] in **c-** and **d-**, a range of values close to V_{Min} ∈[0.4,0.93] corresponding to the virtual velocities obtained for the case without a sediment layer (Fig. 2). These simulations show that whether or not trapping at depths ≤ 1m is considered, this does not significantly change the inferred distributions of the mean velocities and thus the residence times in the river.

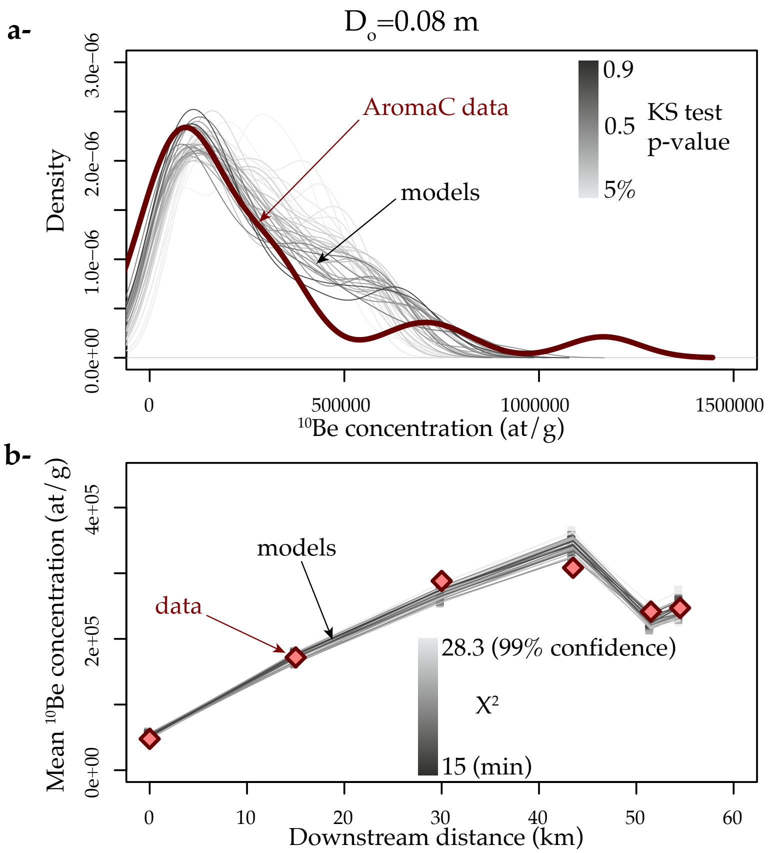

Supplementary Fig. S 8. Same as Fig. 2 but using D_o = 0.08 m instead of 0.14 m. The estimated parameters range from V_{Min} ∈[0.82,1.3] m a^{-1} (instead of [0.4,0.93]) and α ∈[-1.94,-1.28] (instead of [-1.94,-1.04]). This figure illustrates that the confidence intervals of V_{Min} and α do not critically depend on D_o.

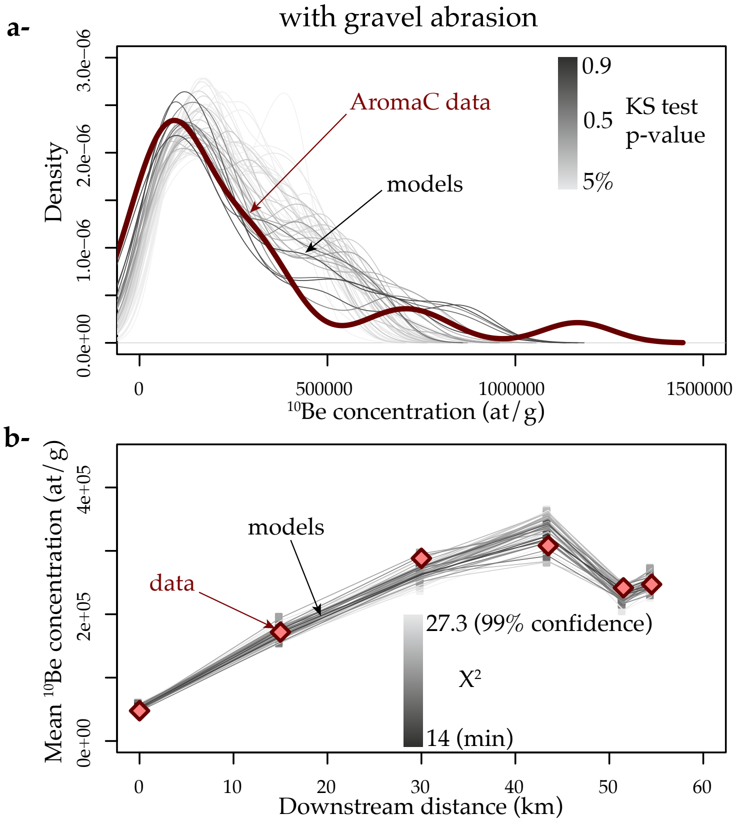

Supplementary Fig. S 9. Same as Fig. 2 but gravel can erode[2] according to the travel distance x using $D = D_{initial}\, e^{-kx}$ with the abrasion rate $k = 10^{-5}$ m^{-1}, an upper value for gneiss[3]. The estimated parameters range as $V_{Min} \in$[0.53,1.1] m a^{-1} (instead of [0.4,0.93]) and $\alpha \in$[-1.93,-1.08] (instead of [-1.94,-1.04]). This simulation shows that the size reduction of gravel by abrasion does not critically change the confidence intervals of V_{Min} and α.

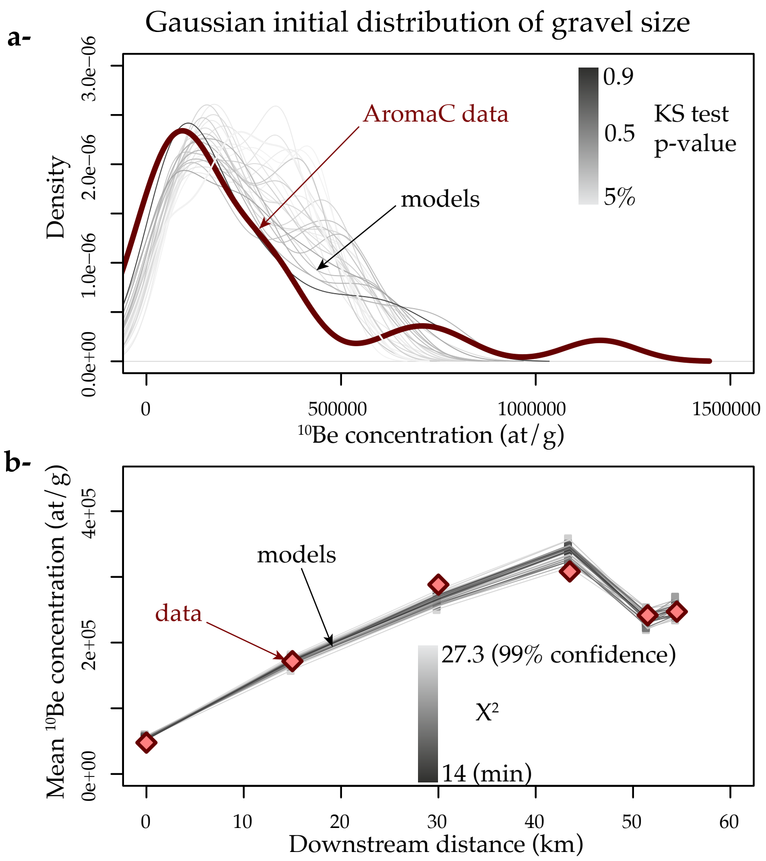

Supplementary Fig. S 10. Same as Fig. 2 but the initial distribution of gravel diameters D entering the river is a Gaussian distribution of mean = 0.15 m and standard deviation = 0.15 m. The p-value of acceptable models is < 0.4 and thus lower on average than in Fig. 2 using a lognormal distribution of D, but the estimated parameters are not significantly modified: $V_{Min} \in$[0.63,0.94] m a^{-1} (instead of [0.4,0.93]) and $\alpha \in$[-2.02,-1.38] (instead of [-1.94,-1.04]). This simulation shows that another distribution of initial D do not change critically the confidence intervals of V_{Min} and α.

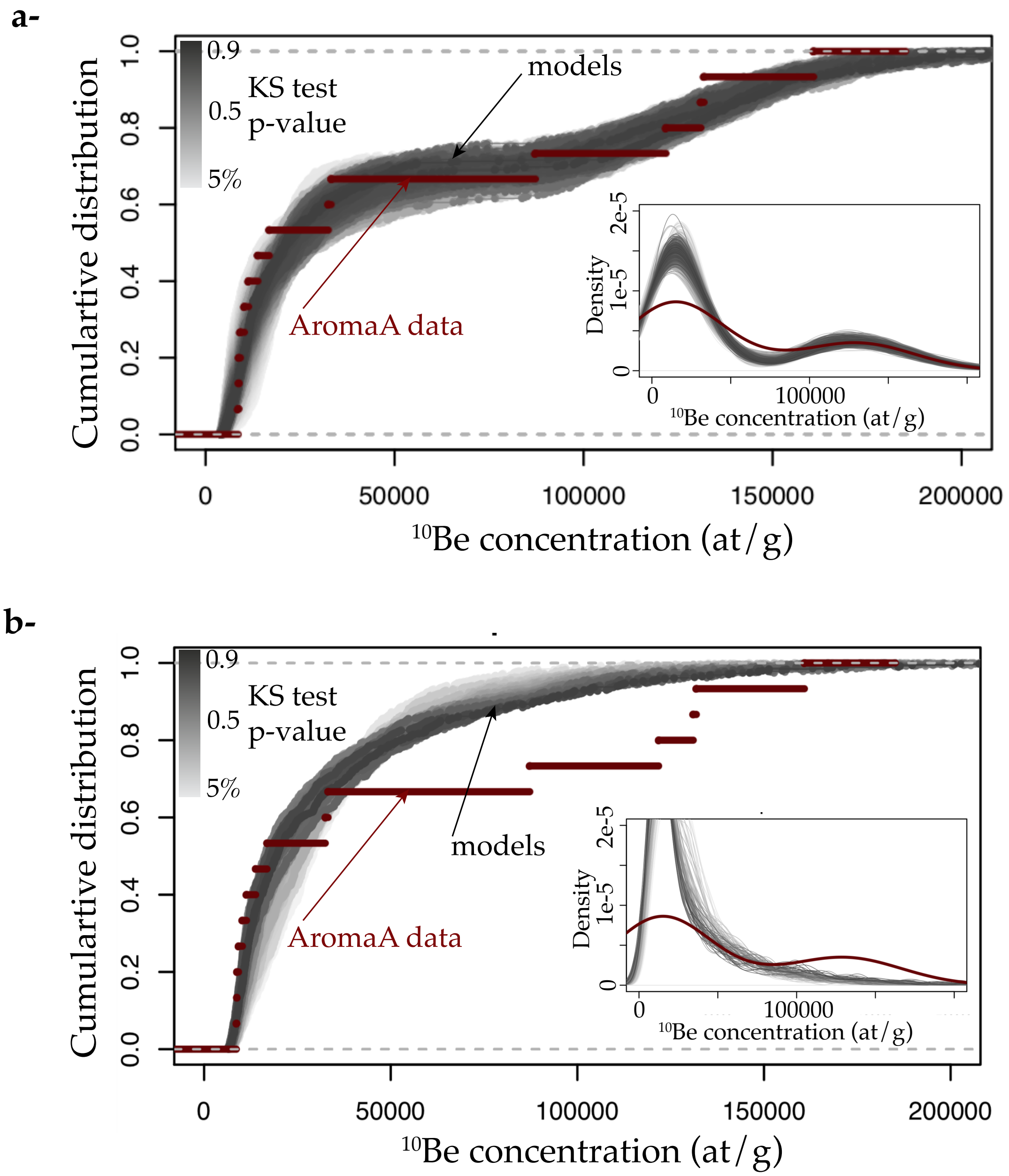

Supplementary Fig. S 11. Estimation of landslide (hillslope) erosion distribution parameters H_{min} and β (see Methods Eq. 5). Gravel size D follows a lognormal distribution of log-mean 0 and log-σ 1.5 between D =0.02 and 0.72 m corresponding to the range of sampled gravel. (**a-**) 70% of clasts are produced by landslides at a mean erosion rate of 1 mm a^{-1} and 30% by detachment from the bedrock with a mean erosion rate of 0.17 mm a^{-1}. The panel compares the cumulative distribution of the AromaA ^{10}Be concentrations and models generated with H_{min} $\in$[0.1,1.5] m and β $\in$[1.1,2.9]. The inset panel shows the density distribution of the ^{10}Be concentrations and highlights the two peaks corresponding to the two populations of clasts produced by either landslides (peak on the left) or bedrock detachment (peak on the right). (**b-**) Same, but 100% of the clasts are produced by landslides at a mean erosion rate of 0.6 mm a^{-1}.
